# Cell size induced bias of current density in hypertrophic cardiomyocytes

**DOI:** 10.1080/19336950.2024.2361416

**Published:** 2024-06-05

**Authors:** Elena Lilliu, Benjamin Hackl, Eva Zabrodska, Stefanie Gewessler, Tobias Karge, Jessica Marksteiner, Jakob Sauer, Eva M. Putz, Hannes Todt, Karlheinz Hilber, Xaver Koenig

**Affiliations:** a Department of Neurophysiology and Neuropharmacology, Center for Physiology and Pharmacology, Medical University of Vienna, Vienna, Austria; b Institute of Anatomy, First Faculty of Medicine, Charles University, Prague, Czech Republic; c Ludwig Boltzmann Institute for Cardiovascular Research at the Center for Biomedical Research and Translational Surgery, Medical University of Vienna, Vienna, Austria; d Institute of Pharmacology, Center for Physiology and Pharmacology, Medical University of Vienna, Vienna, Austria

**Keywords:** Ventricular and Purkinje myocyte, L-type calcium and sodium current density, cell capacitance, cell size, trans-aortic constriction induced hypertrophy, bias

## Abstract

Alterations in ion channel expression and function known as “electrical remodeling” contribute to the development of hypertrophy and to the emergence of arrhythmias and sudden cardiac death. However, comparing current density values – an electrophysiological parameter commonly utilized to assess ion channel function – between normal and hypertrophied cells may be flawed when current amplitude does not scale with cell size. Even more, common routines to study equally sized cells or to discard measurements when large currents do not allow proper voltage-clamp control may introduce a selection bias and thereby confound direct comparison. To test a possible dependence of current density on cell size and shape, we employed whole-cell patch-clamp recording of voltage-gated sodium and calcium currents in Langendorff-isolated ventricular cardiomyocytes and Purkinje myocytes, as well as in cardiomyocytes derived from trans-aortic constriction operated mice. Here, we describe a distinct inverse relationship between voltage-gated sodium and calcium current densities and cell capacitance both in normal and hypertrophied cells. This inverse relationship was well fit by an exponential function and may be due to physiological adaptations that do not scale proportionally with cell size or may be explained by a selection bias. Our study emphasizes the need to consider cell size bias when comparing current densities in cardiomyocytes of different sizes, particularly in hypertrophic cells. Conventional comparisons based solely on mean current density may be inadequate for groups with unequal cell size or non-proportional current amplitude and cell size scaling.

## Introduction

Cardiac hypertrophy is a hallmark of various cardiovascular pathologies [1–3] and is intricately linked to disruptions in ion homeostasis and regulatory mechanisms including ion channel activity [1,4–9]. Alterations in ion channel expression and function known as “electrical remodeling” not only contribute to the development of hypertrophy but also precipitate the emergence of arrhythmias and sudden cardiac death [3,10,11].

Major ionic currents determining the cardiac action potential have been shown to participate in the electrical remodeling during hypertrophy development, including ICaL, INa, Ito, and IK1 [6,8,12,13]. Divergent findings regarding ICaL regulation – ranging from upregulation to downregulation or no discernible change – across different models of hypertrophy underscore the complexity of the underlying phenomena [8,12] and the difficulty of an unambiguous interpretation.

An electrophysiological parameter commonly utilized to assess ion channel function is current density, where current amplitude is divided by cell capacitance, which is a measure of cell size [1]. This ratio must be considered with caution when current amplitude does not scale proportionally with cell size during hypertrophy [14]. Further, common routines to study only equally sized cells (when comparing wildtype to hypertrophic cells, e.g. [12]) or to discard measurements when large currents do not allow proper voltage-clamp control may introduce a selection bias and confound any such direct comparisons.

Here, we investigated the impact of cardiomyocyte size and shape, assessed from physical dimensions and cell capacitance measurements, on current density values of INa and ICaL. To investigate a potential impact across the whole spectrum of cardiomyocyte sizes and shapes, we utilized cardiomyocytes from the ventricles as well as from the Purkinje system. In addition, we employed a model of trans-aortic constriction induced hypertrophy [15], which exhibits maximal hypertrophy at the single cardiomyocyte level. Our findings reveal a distinct inverse relationship between cell capacitance and current density for INa and ICaL in normal and hypertrophied cells.

## Materials and methods

### Animal model and transverse aortic constriction (TAC) operations

Ten male C57BL/6 mice were acquired from Charles River, Germany, specifically opting for the C57BL/6N sub-strain due to its heightened susceptibility to TAC operations [16,17]. Animal housing, the applied TAC surgery procedure and echocardiographic evaluation were described in detail in Hackl et al. [18]. Importantly, we used 6–0 silk (Perma-Hand™, K802H; Ethicon, Johnson & Johnson Medical N.V., Belgium) for aortic constriction and tightened the banding to a 27-gauge needle.

### Cell isolation

Ventricular cardiomyocytes were isolated from the hearts of mice using a Langendorff setup (Hugo Sachs Elektronik, March, Germany). Isolation was performed as previously described [19]. Ventricular myocyte transmural heterogeneity was not accounted for. Cardiac Purkinje myocytes were isolated from a transgenic mouse line (Cx40eGFP/+; [20]) expressing eGFP under the control of the connexin 40 gene, which is specifically expressed in the conduction system of the murine heart. The isolation procedure was identical to the isolation procedure for ventricular cardiomyocytes [19], except an additional enzyme digestion step. The full protocol is detailed in Ebner et al. [21]. Briefly, hearts were rapidly excised, and a cannula was inserted into the aorta for retrograde perfusion with Ca-free solution containing 0.17 mg/ml Liberase TH (Roche) at 37°C for 10 min. Thereafter, the ventricles were cut into pieces and incubated on a shaker at 37°C. Subsequently, the Ca concentration was increased to 150 μM over 30 min in four steps. Pieces of digested ventricular tissue were then triturated to liberate ventricular cardiomyocytes. After a centrifugation step, the myocytes were resuspended in Minimum Essential Medium alpha (Sigma), containing insulin-transferrin-selenium (ITS) media supplement (Sigma) diluted 1:100, 2 mM l-glutamine, 100 u/ml penicillin, 0.1 mg/ml streptomycin, and 17 μM blebbistatin (Sigma). Myocytes were finally plated on Matrigel (Becton Dickinson)-coated culture dishes and were used for confocal microscopy and whole cell patch clamp experiments within six hours after preparation.

### Evaluation of cardiomyocyte size

Isolated single cardiomyoctes were plated onto glass bottom dishes and imaged on a confocal microscope system (Nikon A1R+; 4 images per dish, 2 dishes per mouse heart). Cell size was evaluated by drawing ROIs of fully visible cardiomyocytes using the NIS Elements software (Nikon). Length (l) and width (w) were calculated from ROI-derived surface area and perimeter length assuming a rectangular morphology of the cardiomyocytes, i.e. surface area = l*w and perimeter = 2(l+w) using a custom-written R script.

### Whole cell patch clamp

Individual cardiomyocytes and Purkinje myocytes were patch-clamped on the day of cell isolation using an Axopatch 200B or 700B patch clamp amplifier (Axon Instruments, Union City, CA) in the whole-cell voltage-clamp mode. Capillaries were pulled from aluminosilicate glass (A120-77-10; Science Products, Hofheim, Germany) with a *p*-97 horizontal puller (Sutter Instruments, Novato, CA) to a resistance ranging between 1.0 and 1.5 MΩ when filled with the respective recording solutions. Data acquisition utilized pClamp 11.0 software (Axon Instruments) through a 16-bit A-D/D-A interface (Digidata 1440 or 1550; Axon Instruments). Data analysis was conducted using Clampfit 10.7.0.3 software (Molecular Devices, LLC.) and custom MATLAB and R scripts. Myocyte capacitance values were derived at the beginning of each measurements using the “membrane test” protocol of the Clampex software, which applies a small rectangular voltage step and derives the equivalent circuit parameters, Rs, Rm, and Cm, from an exponential fit to the whole cell capacitive current.

L-type Ca^2+^ current (ICaL) measurements were performed on ventricular cardiomyoctyes of male C57BL/6N SHAM- and TAC-operated mice. External and internal solutions contained, extern.: 5 mM CsCl, 5 mM Glucose, 15 mM HEPES, 2 mM CaCl_2_, 150 mM NMDG, pH 7.4 with HCl; intern.: 102 mM CsCl, 10 mM HEPES, 5 mM MgCl_2_, 5 mM Na_2_ATP, 10 mM TEA-Cl, 10 mM EGTA, pH 7.4 with CsOH). L-type Ca^2+^ currents were elicited by rectangular voltage steps of 200 ms length from −80 to 50 mV in 5–10 mV increments from a holding potential of −80 mV.

Measurements of Na^+^ currents (INa) were performed as previously described [22]. Briefly, ventricular cardiomyocytes were isolated from male C57BL/10 ScSnJ mice and Purkinje myocytes from male C57BL/10 ScSnJ-CX40^eGFP/+^ mice [20]. Recordings were made in a bath solution that consisted of (in mM): 5 NaCl, 135 N-methyl-D-glucamine, 2.5 KCl, 1 CaCl2, 1 MgCl2, 10 HEPES; pH = 7.4, adjusted with HCl. The bath solution additionally contained 17 μM blebbistatin. The pipette solution contained (in mM): 5 NaCl, 110 CsF, 10 EGTA, 10 HEPES; pH = 7.3, adjusted with CsOH. Membrane voltages were corrected for liquid junction potentials. Sodium currents were elicited from a holding potential of −117 mV by 25 ms rectangular voltage steps to potentials between −97 and −7 mV. Maximal inward current was divided by cell capacitance to yield current density values. Current-voltage (I-V) relationships were fit with the function I = G_max_·(V-V_rev_)/(1+exp((V_50_-V)/k)), where I is the current, G_max_ is the maximum conductance, V is the membrane potential, V_rev_ is the reversal potential, V_50_ is the voltage at which half-maximum activation occurred, and k is the slope factor. To derive the voltage-dependence of steady-state inactivation myocytes were held at a potential of −127 mV. An inactivating pre-pulse of 50 ms duration was applied to different potentials between −127 and −27 mV before applying a 25 ms test pulse. Maximal inward current amplitudes during the test pulse were plotted over pre-pulse voltages to obtain the voltage-dependence of inactivation. Respective data were normalized and fit with a Boltzmann function, I_norm_ = 1/(1+exp((V−V_50_)/k)), with I_norm_ the normalized current, V the membrane potential, V_50_ the voltage at which half-maximum inactivation occurred and k the slope factor.

Statistical analysis was performed with Graph Pad Prism software. Two groups were compared with unpaired Students *t*-test or Mann–Whitney *U* test when normally or non-normally distributed, respectively. Normality was tested using the D’Agostino-Pearson omnibus test. *p* < 0.05, 0.01, 0.001, 0.0001 is indicated by *, **, ***, and ****.

## Results

**Figure 1.**
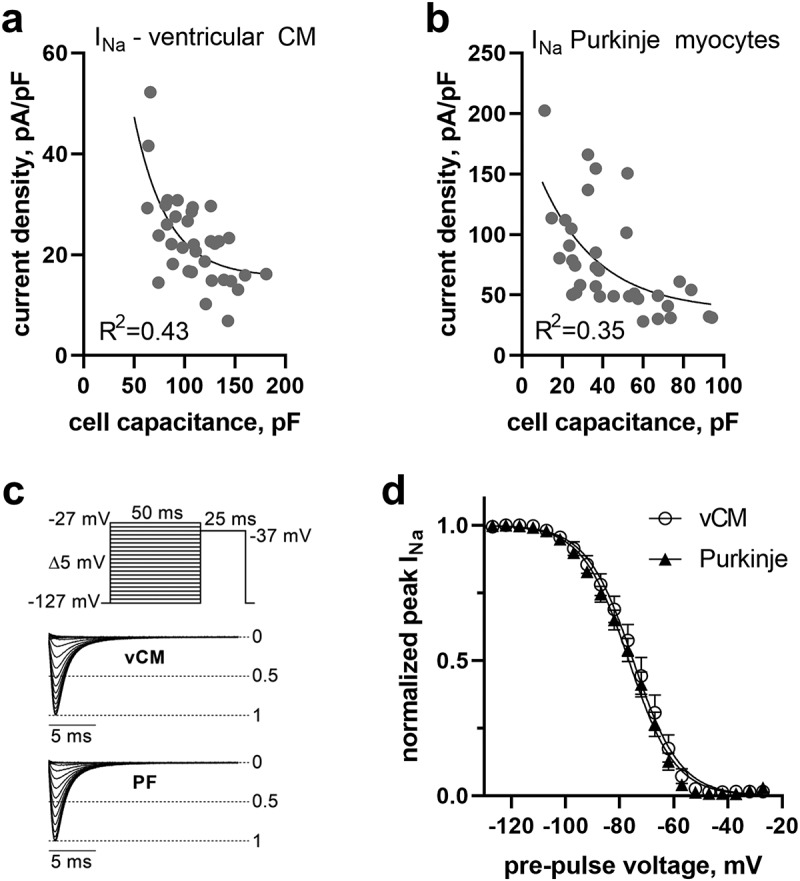
Na current density (INa) relates inversely to cell capacitance in mouse ventricular and Purkinje myocytes. INa density plotted against whole-cell capacitance values of individual ventricular (a) and Purkinje (b) myocytes isolated from the hearts of wildtype mice. (c) Voltage-clamp protocol and normalized original INa traces in ventricular (vCM) and Purkinje (PF) myocytes to test for steady-state voltage-dependence of fast inactivation. (d) Steady-state voltage dependence of fast inactivation for ventricular and Purkinje myocytes. Boltzmann function fit values for the half point of inactivation (V_50_) amounted to −75 and −77 mV and for the corresponding slope factor (k) to −8.6 and −8.6 mV, respectively.

Cardiomyocytes from the ventricles and the Purkinje system were obtained from the hearts of C57BL/6 mice using a Langendorff isolation. Voltage-gated sodium (Na) current (INa) amplitudes were recorded in whole-cell patch clamp mode and current density values were calculated by dividing maximal INa by the cell capacitance, an electrophysiological measure of cell size. When plotting individual current density values of each cell over respective capacitance values, we noticed a strong inverse relationship in cardiomyocytes isolated from the ventricles (Figure 1a); larger cells had smaller current densities compared to smaller cells. We asked if the size and specific physical shape of cardiomyocytes and the expressed Na channel density might contribute to the observed phenomenon. To this end, we measured INa in cardiac Purkinje myocytes, which are known to have higher Na channel density and are longer in shape [23]. A very similar relationship was observed in this dataset (Figure 1b). Notably, full availability of sodium channels was ensured by the chosen holding potential of −117 mV in our recordings of INa in ventricular and Purkinje myocytes (Figure 1c,d).

**Figure 2.**
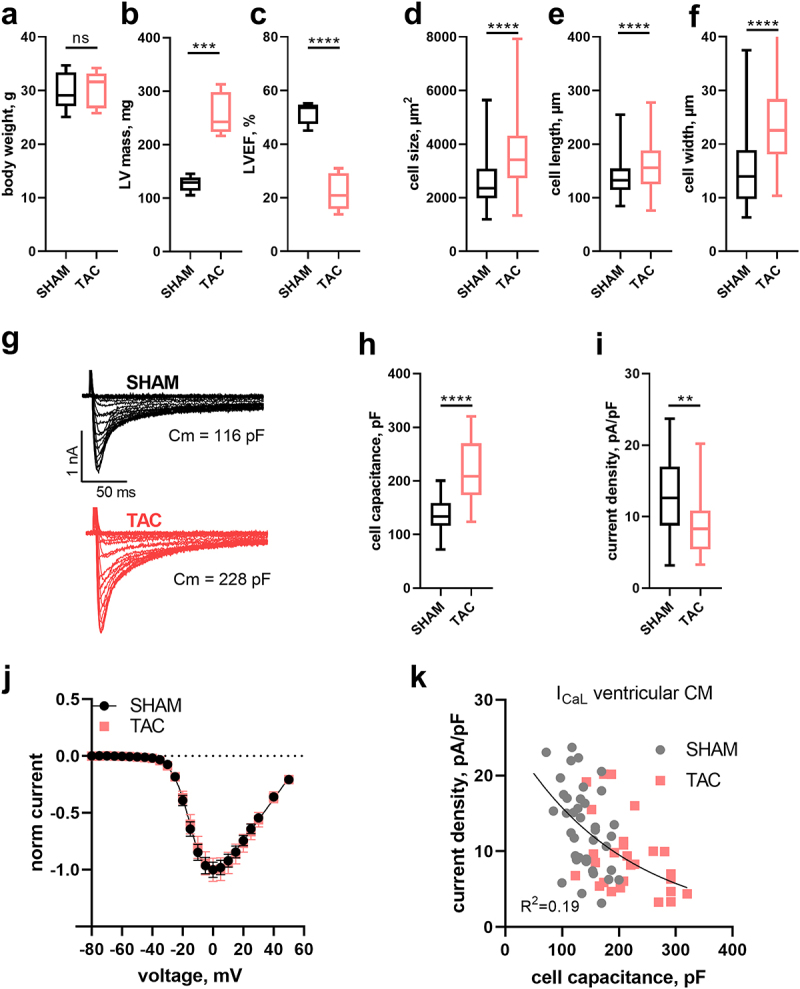
Ca current (ICaL) density relates inversely to cell capacitance in ventricular myocytes of SHAM- and TAC-operated mice. Box (25^th^ to 75^th^ percentiles) and whiskers (min to max) plot of body weight (a), left-ventricular mass (LVmass, b) and left-ventricular ejection fraction (LVEF, c) of SHAM- and TAC-operated mice and of isolated myocyte surface area (cell size, d), cell length (e) and width (f) of respective animals. (g) Typical original ICaL traces recorded in the whole-cell patch-clamp configuration during different depolarizing voltage steps of − 80 to +50 mV from a holding potential of −80 mV. Cell capacitance (Cm) of respective cells is indicated. Box and whiskers plot as above of cell capacitance (h) and ICaL density (i) from n = 40 (5) and n = 27 (5) myocytes of SHAM and TAC-operated mice, respectively. The number of animals is given in brackets. (j) ICaL to voltage relationship (mean ± SEM) of myocytes plotted in h and i. (k) ICaL density to cell capacitance plot of myocytes from normal (SHAM-operated; gray) and hypertrophied (TAC-operated; red) hearts. Solid line represents a single exponential fit to the data that is not statistically different between SHAM and TAC. R^2^ of the fit is indicated.

Next, we wanted to elucidate if this finding was specific for INa or would also apply to the L-type calcium (Ca) current (ICaL), and if the inverse relationship would be enhanced during cardiac hypertrophy. To this end, we performed TAC-operations on five male C57BL/6 mice to induce cardiac pressure overload-induced hypertrophy and heart failure [15,18] and SHAM operations of five male mice of the same strain. Eight weeks after TAC mice showed unaltered body weight (Figure 2a) but a significant increase in left-ventricular mass (LVmass; Figure 2b) and a reduction in left-ventricular ejection fraction (LVEF; Figure 2c) in agreement with the reported phenotype of this model [16–18]. Hypertrophy was also evident at the cellular level in individual cardiomyocytes, which exhibited increased size (Figure 2d), length (Figure 2e) and width (Figure 2f). We then performed whole-cell patch-clamp experiments to assess a possible regulation of L-type Ca channels in hypertrophied cardiac myocytes. Figure 2g shows typical whole-cell recordings of ICaL elicited by rectangular voltage-steps to different potentials (see methods for details) in a cardiomyocyte isolated from a SHAM- and TAC-operated animal, respectively. Although the hypertrophied cell (isolated from the TAC-operated animal) was almost twice as large, as indicated by the provided cell capacitance values (Figure 2g), respective current amplitudes did not scale accordingly, i.e. the amplitude was not twice as large (compare lower and upper trace). To quantify, we determined the capacitance and calculated the current density of all measurments. Owing to the hypertrophy, single myocyte capacitance values were significantly increased in TAC- versus SHAM- derived cardiomyocytes (Figure 2h), consistent with evaluated myocyte physical dimensions (Figure 2d–f) and left ventricular mass (Figure 2b) derived from echocardiography. When comparing current density values derived from cardiomyocytes of SHAM and TAC animals a significant reduction was observed (Figure 2i). The voltage-dependence of activation was not different in cardiomyocytes of SHAM- compared to TAC-operated animals (*p* = 0.8, Extra sum-of-square *F*-test), as indicated by overlapping curves when comparing normalized IV-relationships (Figure 2j); respective fit values are given in Table 1. This confirms recent reports [8] and indicates proper voltage-clamp in hypertrophied cells, as otherwise respective relationship would typically be shifted toward more hyperpolarized potentials and the steepness of activation would increase. When plotting ICaL density values against cell capacitance, we observed an inverse relationship that was well fit with a single decaying exponential (Figure 2k). Despite the myocyte capacitance being larger and current density values smaller in cardiomyocytes of TAC compared to SHAM operated mice, single exponential model fits to both data sets did not differ statistically from each other. Hence, a common model was to be preferred (*p* > 0.75, extra sum-of-squares *F*-test). This suggested that the relationship between ICaL density and cell capacitance was the same for SHAM- and TAC-derived cardiomyocytes, but that current density values were skewed toward smaller values in larger cells.

**Table 1.** Best fit values for the mean L-type Ca current-voltage relationships in myocytes derived from SHAM- and TAC-operated animals. Values are given as best fit value and 95% confidence interval.

	SHAM		TAC	
	Best fit	95% CI	Best fit	95% CI
V_rev_, mV	61	[57, 65]	60	[55, 67]
V_50_, mV	−14	[−15, −13]	−13	[−15, −10]
K, mV	5.8	[5.0, 6.6]	6.2	[5.0, 7.6]

## Discussion

The observed inverse relationship between current density and cell capacitance might originate from the existence of different pools of channels in different regions of a cardiomyocyte. For INa, it has been shown that a major pool of Na channels is expressed at the intercalated discs while other Na channel clusters are located at the lateral membrane and the t-tubular system [24,25]. Indeed, these populations of ion channels have been shown to vary in functional properties [26]. The relative fractions of these surface areas, i.e. the intercalated disc, lateral membrane and t-tubular system, may change with cardiomyocyte size and hence may account for reduced current density values if regions of high channel density are underrepresented in large cells. In line, previous studies demonstrated that ventricular hypertrophy is associated with a decline in longitudinal conduction velocity and an increase in transverse conduction velocity [27]. Such changes may result from alterations of the relative distribution of Na channels in lateral membrane versus the intercalated disc regions. Perhaps such hypertrophy-induced modulation of local Na channel distribution is reflected in the cell size-dependent differences in INa identified in this study. Notably, while Na channel proteins can be detected by immunofluorescence techniques within the t-tubular system, these channels do not contribute significantly to whole-cell INa [28,29]. Thus, whole cell INa did not decrease after detubulation, strongly indicating that Na channels at the intercalated disk and the lateral membrane but not from within the t-tubular system conduct the vast majority of INa, or that INa occurring within the t-tubular system is not detected in whole-cell patch-clamp experiments [28,30,31].

Another point of consideration is a potential transmural heterogeneity of Na channel availability. Remme et al. [32] have reported that subepicardial mouse myocytes showed reduced Na channel availability compared to subendocardial myocytes. We did not discriminate between these myocyte populations in our isolation procedure. Thus, such heterogeneity could possibly account for the inverse relationship between current density and cell capacitance values if isolated endocardial myocytes happen to be consistently larger than epicardial myocytes. While we are not aware that such systematic differences in size exist in different regions of the working murine myocardium, we acknowledge that the presence of multiple populations of myocytes in our experiments certainly adds a degree of uncertainty and may be considered a limitation of our study.

L-type Ca channels are predominantly expressed in the t-tubular system [33], which undergoes substantial remodeling during hypertrophy development [34]. Specifically, t-tubules degrade and are increasingly lost. A reduction of the t-tubular system area and of L-type Ca channels residing therein is therefore expected to result in a reduction of current density. Indeed, it has been reported that ICaL density is decreased in severe hypertrophy [8,12], and this finding accords with the evaluation of current density mean values in our study (Figure 2i). However, our transmitted light images that were used for evaluation of cell size and shape did not show any obvious structural deterioration of cardiomyocytes, suggesting that substantial remodeling of the t-system did not occur under our experimental conditions.

While some previous studies have reported an increased ICaL density in severe hypertrophy, others have reported no change or even a decrease [8,12]. Indeed, a more detailed analysis of our data (Figure 2k) suggested that current density was in fact not altered in TAC- compared to SHAM-derived cardiomyocytes, but that current density values merely inversely correlated with cell size. An inverse relationship between Ca current density and cell capacitance has been reported before [35]. Importantly however, and extending the report of Nuss et al. [35], we demonstrate that this relationship was seen in SHAM- and TAC-derived cardiomyocytes independently. This suggests that the relationship is independent of hypertrophy development per se unless a reduction of ICaL density also occurs under “physiological” hypertrophy in normal cells when the number of channels remains constant despite an increase in cell size [1]. It may also suggest that alterations in t-tubular structure and potential concomitant reduction of ICaL are unlikely to account for the observed relationship.

As we observed an inverse relationship between current density and cell capacitance for both INa (Figure 1) and ICaL (Figure 2), this may suggest a methodological issue. Thus, a bias could potentially emerge from larger current amplitudes in larger cells, which may not be properly voltage-clamped and hence excluded from subsequent analysis. Consequently, results would be biased toward cells with smaller current amplitudes and hence smaller current density values. In that light the observed reduction of current density in hypertrophied cells may only be an apparent one and reflect a simple selection bias. To avoid such bias only myocytes of equal size may be evaluated, as in, e.g. [35]. However, restricting measurements to equally sized myocytes will exclude large cells with overt hypertrophy and thereby potentially exclude those cells with the most pronounced phenotype. Specific changes occurring as a result of hypertrophy might therefore be overlooked when studying only cardiomyocytes of “normal” size. Rather than to confine cell capacitance values, an alternative way to analyze such data is to plot current density against cell capacitance values (Figure 2k) and then test if respective model descriptors (we chose an exponential decay) are different across tested groups. This strategy has also been employed by others [14,36]. Another possible solution would be to limit current amplitudes to a certain range, accounting for changes in cell size and during hypertrophy or to reduce current amplitudes by designing recording solutions with reduced concentrations of the conducting ion [37]. Finally, another evaluation paradigm may be provided by the cell attached patch mode of recordings, whereby not the whole cell but only a small membrane patch is measured. While this technique has been employed successfully in the last years to decipher the localization and density of Nav1.5 channels in ventricular cardiomyocytes [13,25], the predominant localization of L-type Ca channels within the t-tubular system [33] excludes those channels to be studied by the same approach.

In summary, our study highlights the necessity to consider myocyte size bias when comparing current density values in cardiomyocytes of different sizes. This finding is particularly relevant when investigating differences between normal and hypertrophic cardiomyocytes where myocyte size is systematically different. Conventional comparisons using current density mean values only may fail for groups of unequal myocyte size or when current amplitude and myocyte size do not scale proportionally.

## Data Availability

The data that support the findings of this study are available from the corresponding author, Xaver Koenig, upon reasonable request.
